# Comparing the EQ-5D-5L utility index based on value sets of different countries: impact on the interpretation of clinical study results

**DOI:** 10.1186/s13104-019-4067-9

**Published:** 2019-01-14

**Authors:** Christoph Gerlinger, Luke Bamber, Friedhelm Leverkus, Carsten Schwenke, Claudia Haberland, Gilda Schmidt, Jan Endrikat

**Affiliations:** 1 Statistics and Data Insights, Bayer AG, Müllerstr 178, 13353 Berlin, Germany; 2 Obstetrics and Gynecology, Saarland University, Homburg, Saar Germany; 3 Health Economics and Outcomes Research, Bayer AG, Wuppertal, Germany; 4 Health Economics and Outcomes Research, Pfizer Deutschland GmbH, Berlin, Germany; 5 SCOSSIS, Berlin, Germany; 6 Radiology, Bayer AG, Berlin, Germany

**Keywords:** EQ-5D-5L utility index, Clinical studies, Country differences, Quality of life valuation

## Abstract

**Objective:**

To compare the country-specific value sets of the EQ-5D-5L utility index and to evaluate the impact on the interpretation of clinical study results. Six country value sets from Canada, England, Japan, Korea, Netherlands and Uruguay were obtained from literature. In addition, ten crosswalk value sets were downloaded from the EuroQol.org website.

**Results:**

For each of the 3125 possible health states the difference between the country with the highest index and the country with the lowest index was calculated. The median difference was 0.417 across the health states. When analyzing multinational clinical studies, country-specific value sets should be used to evaluate treatment effects. Additional country-specific analyses are needed.

**Electronic supplementary material:**

The online version of this article (10.1186/s13104-019-4067-9) contains supplementary material, which is available to authorized users.

## Introduction

In 1990, the EuroQol Research Foundation, a non-profit international network of international multidisciplinary researchers, developed a health status measuring tool called ‘EuroQol EQ-5D-3L’ with 5 dimensions and 3 levels of severity [1]. Today it is one of the most frequently used generic questionnaires to assess the patients’ health states and estimate utilities. These utilities are necessary to calculate quality adjusted life years (QALYs) [2, 3] a widely used measure in health technology assessment. In 2005 a new version with five levels of severity (EQ-5D-5L) was introduced, replacing the initial three level of severity (EQ-5-D-3L) version in order to improve the instrument’s sensitivity. The EQ-5D-3L has been translated in 170 languages, the EQ-5D-5L in up to 130 languages; both versions are available in different modes of administration and have been used worldwide. EQ-5D health states may be directly converted into country-specific single index values (utilities) using country specific value sets. For the new 5-level version elucidated value sets are available for six countries. Alternatively, for ten countries so called ‘crosswalk’ value sets are available. These enable estimation of utilities for EQ-5D-5L based on the existing value sets for the EQ-5D-3L.

The goal of this study was to compare the country-specific value sets of the EQ-5D-5L utilities and to evaluate the impact on the interpretation of clinical study results in the general population.

## Main text

### Methods

The EQ-5D is a generic, standardized and simple health related quality of life instrument for clinical and economic appraisal, applicable to a wide range of conditions and treatments [4]. Patients complete the simple questionnaires either during face-to-face interviews, on electronic devices, online or, alternatively, submit their responses as a hard copy. This instrument is intellectually non-demanding and it takes just a couple of minutes to complete [4].

The EQ-5D-5L descriptive system of health states comprises 5 dimensions (‘5D’): (1) mobility; (2) self-care; (3) usual activities; (4) pain/discomfort and (5) anxiety/depression. Those are rated by a verbal 5-point rating scale allowing for distinction of five levels (‘5L’) of severity: Level 1: no problems; Level 2: slight problems; Level 3: moderate problems; Level 4: severe problems; Level 5: extreme problems per dimension and providing a 1-digit number for each dimension. The digits for the 5 dimensions can be combined in a 5-digit code describing the patient’s health state. A total of 3125 combinations and therewith different health states are possible. These may be converted into a country-specific single index value (e.g. preference weight, preference-based value, utility, QALY weight) using country specific value sets, which have been derived from large country-specific validation studies using time-trade-off/discrete choice methodology [5] and which anchor 1 for ‘perfect health’ and 0 for ‘dead’, respectively. The single index value can then be used to inform country specific economic evaluations of health care interventions and enable calculation of quality adjusted life years.

To date six country specific value sets for the direct estimation of EQ-5D-5L single index values are available (England, Japan, Canada, Uruguay, Netherlands, Korea) and were obtained from literature [6–11]. In the interim additional EQ-5D-5L country specific single index values can be obtained via the EQ-5D-5L Crosswalk Project. Based on patients’ completion of both the EQ-5D-3L and the EQ-5D-5L descriptive systems, the Crosswalk Project established a link between the EQ-5D-5L and the EQ-5D-3L descriptive system, for which value sets in more countries are available (Belgium, Denmark, Europe, Finland, France, Germany, Japan, Netherlands, New Zealand, Slovenia, Spain, Thailand, UK, US, Zimbabwe). By using the crosswalk link function and the individual responses to the EQ-5D-5L descriptive system, the single index value for the EQ-5D-5L can be estimated. The crosswalk value were downloaded from the EuroQol.org website [1].

The evaluations of health states were compared between two countries. For each of the 3125 different health states the difference between the two countries with the highest and the lowest value was calculated. These differences were analyzed by descriptive statistics including boxplots and histograms. The differences in valuations between two different health states, e.g. changing from health state “12345” to “54321”, were also calculated and analyzed for each pair of distinct health states and for each country.

An analysis of variance with factors health state and country was used to exploratively test the null hypothesis of no country differences for the health states.

Boxplots were drawn using 1.5 times the interquartile range as the maximal length of the whiskers. The correlation of the values between any two countries was analyzed by scatter plots.

To gauge the effects of the different valuations in practice the baseline EQ-5D-5L states of a real study were evaluated for each of the countries. All analyses were performed using SAS version 9.4 software.

### Results

There were substantial differences in the utility index between countries in the values attributed to each health state.

**Fig. 1:**
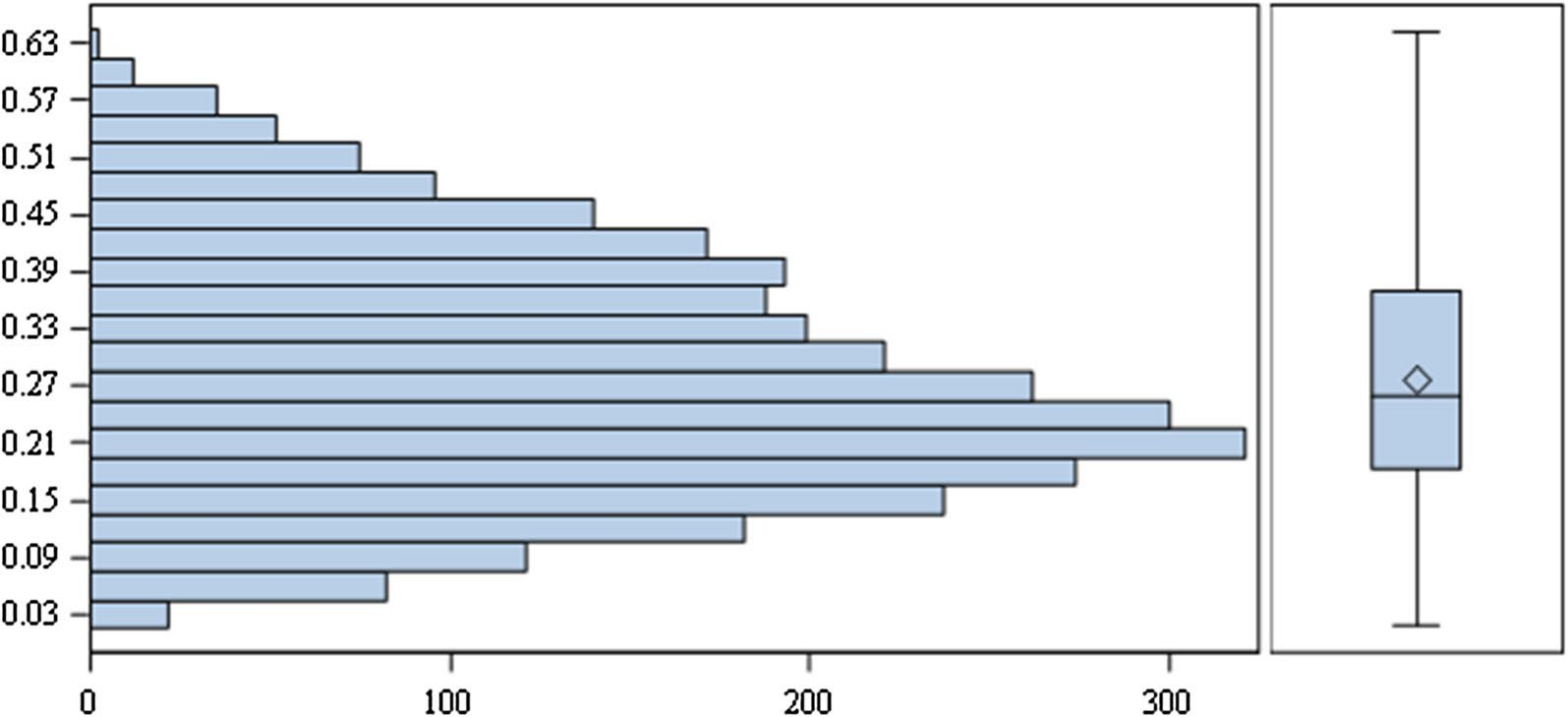
Maximal difference between countries—elicitated sets

**Fig. 2:**
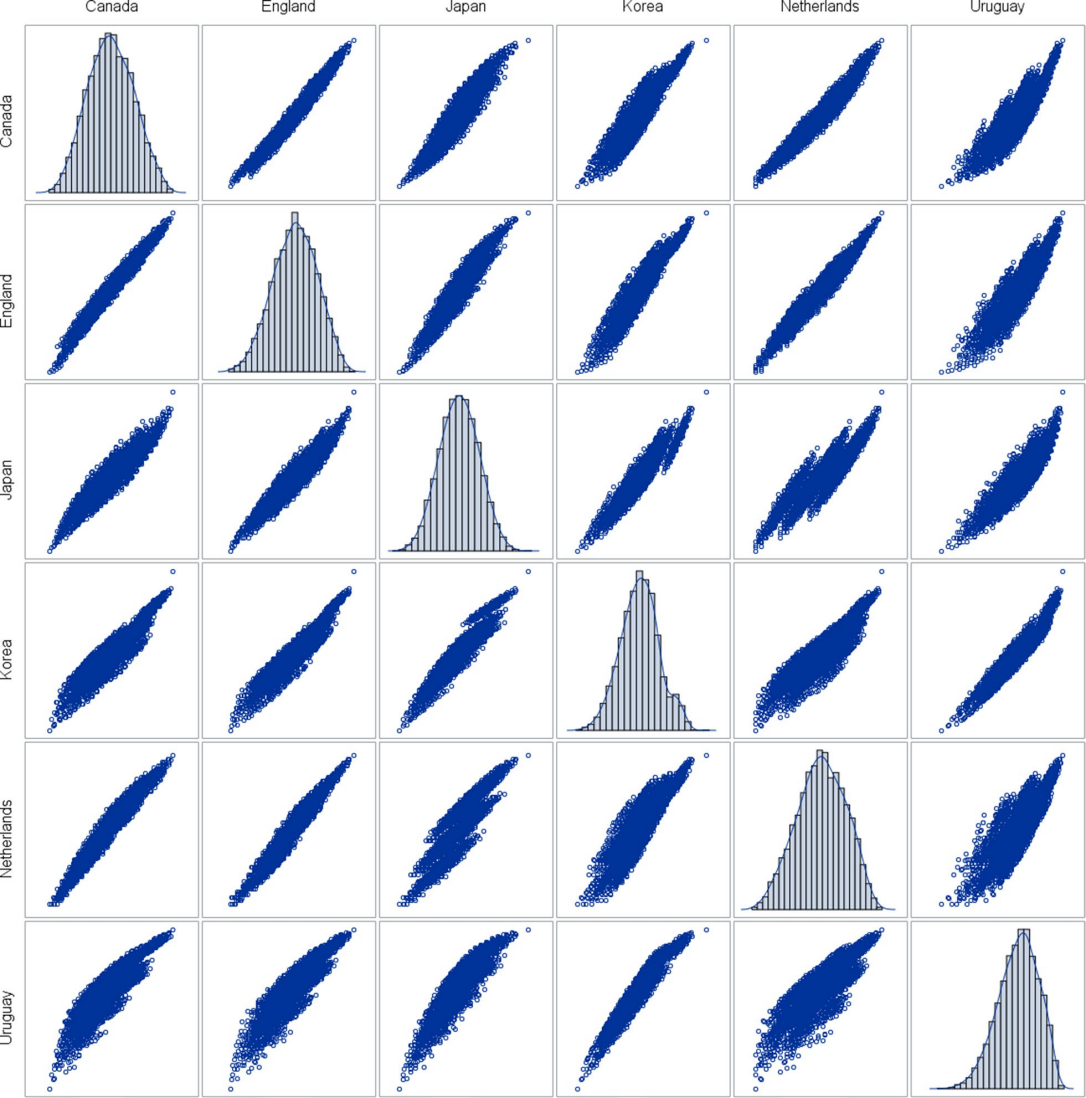
Scatter plot by country—elicitated sets

For the six countries with elicitated value sets the discrepancy between the country with the highest valuation and the country with the lowest valuation of the health state ranged from 0.0173 for health state “45512” to 0.642 for health state “44444”. The latter health state was valued at − 0.289 in The Netherlands and at + 0.353 in Uruguay. The median discrepancy across all 3125 possible health states was 0.260 with an interquartile range (IQR) of 0.182 to 0.371 (Fig. 1). The valuations were significantly (p < 0.0001) different between the countries (Fig. 2).

For the 10 crosswalk value sets the median difference between the country with the highest index and the country with the lowest index was 0.417 across the 3125 possible health states with an IQR of 0.337 to 0.490. The smallest difference was 0.100 for health state “11111” and the highest difference was 0.626 for health state “15155” with Japan scoring +0.440 and the UK scoring − 0.186. For 99% of the health states the difference was larger than 0.190 (Additional file 1: Figure S1). The valuations were significantly different between the countries (p < 0.0001).

There were also systematic differences between countries. E.g., in almost all health states, Germany reported higher valuations than France (Additional file 2 Figure S2).

Large discrepancies between the countries were also observed when analyzing changes from one health state to another health state. In many cases not only the magnitude of the change was different between the countries but also the direction of the change. E.g., a change from health state “44444” to “55511” is valued as an improvement of 0.679 units in The Netherlands and as a worsening of 0.169 units in Uruguay.

For the sample study with 313 patients included the smallest valuation in the six countries with elucidated sets was Japan with a median of 0.717 and the highest valuation was Uruguay with a median of 0.861. For the crosswalk sets the lowest valuation was Thailand with a median of 0.582 and the highest valuation was Germany with a median of 0.806 (Table 1).

**Table 1:** Valuations of sample study using different country value sets

Value set	N	Mean	Std dev	Minimum	Lower quartile	Median	Upper quartile	Maximum
Canada	313	0.698	0.208	0.019	0.572	0.765	0.860	0.949
England	313	0.713	0.229	− 0.117	0.558	0.786	0.893	1.000
Japan	313	0.699	0.176	0.161	0.581	0.717	0.831	1.000
Korea	313	0.704	0.173	0.172	0.560	0.765	0.830	1.000
Netherlands	313	0.622	0.273	− 0.289	0.449	0.717	0.848	0.953
Uruguay	313	0.818	0.147	0.240	0.730	0.861	0.927	1.000
Denmark (cw)	313	0.677	0.175	0.083	0.555	0.722	0.797	1.000
France (cw)	313	0.604	0.268	− 0.169	0.402	0.635	0.839	1.000
Germany (cw)	313	0.742	0.202	0.069	0.595	0.806	0.887	1.000
Japan (cw)	313	0.665	0.132	0.127	0.571	0.671	0.740	1.000
Netherland (cw)s	313	0.658	0.212	0.088	0.503	0.713	0.833	1.000
Spain (cw)	313	0.666	0.241	− 0.198	0.509	0.729	0.857	1.000
Thailand (cw)	313	0.570	0.199	− 0.124	0.432	0.582	0.723	1.000
UK (cw)	313	0.610	0.235	− 0.107	0.442	0.696	0.768	1.000
US (cw)	313	0.714	0.158	0.188	0.605	0.777	0.826	1.000
Zimbabwe (cw)	313	0.710	0.131	0.303	0.621	0.743	0.810	0.900

*cw* crosswalk set

### Discussion

The aim of this analysis was to compare country-specific value sets of the EQ-5D-5L utility index and to evaluate the impact on the interpretation of clinical study results.

The utilities of health states vary substantially between the different countries. The median difference between the country with the highest index and the country with the lowest index was 0.417 for the crosswalk sets and 0.315 for the countries with elucidated value sets across all 3125 possible health states. For the sample study the valuations of the patients differed by 0.114 for the countries with elucidated value sets and by 0.224 for the crosswalk sets.

Of course, some differences between the different valuations are to be expected, namely because of different study settings, sampling error, or, cultural, environmental differences or health system differences. E.g., being restricted in mobility or usual activities through physical disability may have different impact on societal valuations of quality of life for, for instance, wheelchair users depending on the both health system support and the prevalence of supportive infrastructure in society in general. The important issue is the magnitude of the observed differences of roughly 1/3 of the patient’s quality of life.

The EQ-5D is validated for several languages and countries. Assuming an adequate similarity of the country-specific versions of the questionnaire, the answers are regarded as sufficiently similar. By the choice of the value set, the analysis is focused on a specific task. Using different value sets within the same analysis lead to very heterogeneous results, which cannot be interpreted if many countries are involved. Therefore, if the utility index is to be used for health economic modelling, the value set of that specific country should, in agreement with the regulating agency, e.g. health technology assessment (HTA), be used to avoid misinterpretation based on the “wrong” value set. For example the French HTA body Haute Autorité de Santé requests the use of the French value set for the EQ-5D [12]. On the other hand, the analysis of a clinical trial that aims for the estimation of the treatment effects may make use of a single value set to calculate treatment differences. The absolute values of the utility index per treatment groups cannot be interpreted as stand-alone, but the difference can. On the other hand, meta-analyses based on several clinical trials may be biased if either the value set is not the same or in case of a different set of countries involved in the trial.

As of today, for e.g. discussion with UK payer organizations (e.g. NICE) in multinational studies usually the value set of England is applied for all other participating countries. As the analysis presented here shows, this leads to false assumptions as the value sets between the countries vary considerably. Country specific differences were addressed in the past [5] but to the best of our knowledge, this is the first time that the magnitude of this difference has been described for the EQ-5D-5L. Today, only 6 country-specific value sets have been published. In order to further elucidate these differences, more country-specific value sets are needed.

### Conclusions

When analyzing multinational clinical studies, country-specific value sets should be used to evaluate treatment effects. Using just one country set, e.g. the one from England, provides results that are only valid for that country. Country-specific analyses are needed for additional countries.

## Limitations

- Elicitated value sets were only available for a few countries.
- The value sets are from a societal perspective only.
- The sample size of the clinical study was limited and we could only analyze data at baseline and no changes over time.

## Additional files

**Additional file 1.** Maximal difference between countries—crosswalk sets.

**Additional file 2.** Scatter plot by country—Crosswalk sets. Denmark = ”1” France = ”2” Germany = ”3” Japan = ”4” Netherlands = ”5” Spain = ”6” Thailand = ”7” UK = ”8” US = ”9” Zimbabwe = ”10”.

## Abbreviations

HTA: health technology assessment
IQR: inter quartile range
QALY: quality adjusted life year

## References

[1] https://euroqol.org/eq-5d-instruments/eq-5d-5l-about/valuation-standard-value-sets/. Accessed 10 Nov 2018 and the references cited therein.

[2] Weinstein MC, Torrance G, McGuire A (2009). QALYs: the basics. Value Health.

[3] Fauteux V, Poder TG (2017). État des lieux sur les méthodes d’élicitation du QALY. Int J Health Prefer Res.

[4] EuroQol Group (1990). EuroQol-a new facility for the measurement of health-related quality of life. Health Policy.

[5] Xie F, Gaebel K, Perampaladas K, Doble B, Pullenayegum E (2014). Comparing EQ-5D valuation studies: a systematic review and methodological reporting checklist. Med Decis Making.

[6] Xie F, Pullenayegum E, Gaebel K, Bansback N, Bryan S, Ohinmaa A, Poissant L, Johnson JA (2016). A time trade-off-derived value set of the EQ-5D-5L for Canada. Med Care.

[7] Augustovski F, Rey-Ares L, Irazola V, Garay OU, Gianneo O, Fernandez G, Morales M, Gibbons L, Ramos-Goni JM (2016). An EQ-5D-5L value set based on Uruguayan population preferences. Qual Life Res.

[8] Devlin N, Shah K, Feng Y, Mulhern B, van Hout B (2016). Valuing health-related quality of life: an EQ-5D-5L value set for England.

[9] Ikeda Shunya, Shiroiwa Takeru, Igarashi Ataru, Noto Shinichi, Fukuda Takashi (2015). Developing a Japanese version of the EQ-5D-5L value set. J Natl Inst Public Health.

[10] Kim SH, Ahn J, Ock M, Shin S, Park J, Luo N, Jo MW (2016). The EQ-5D-5L valuation study in Korea. Qual Life Res.

[11] Versteegh MM, Vermeulen KM, Evers SM, de Wit GA, Prenger R, Stolk EA (2016). Dutch tariff for the Five-Level Version of EQ-5D. Value Health.

[12] de Santé HA (2011). Choix méthodologiques pour l’évaluation économique à la HAS.

