# Supplementary figures and images for: Comparing the EQ-5D-5L utility index based on value sets of different countries: impact on the interpretation of clinical study results
Source: BMC Res Notes. 2019 Jan 14;12:18. doi: 10.1186/s13104-019-4067-9 (PMC6332559; doi:10.1186/s13104-019-4067-9)

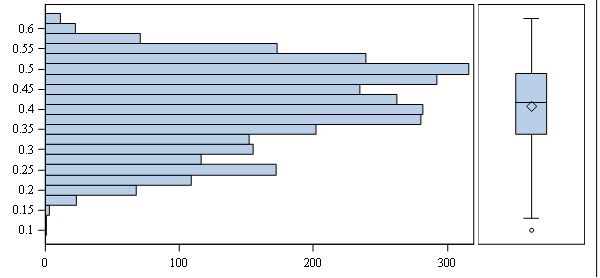

Supplement: Supplementary file 1 — Additional file 1. Maximal difference between countries—crosswalk sets. [file 13104_2019_4067_MOESM1_ESM.tif]

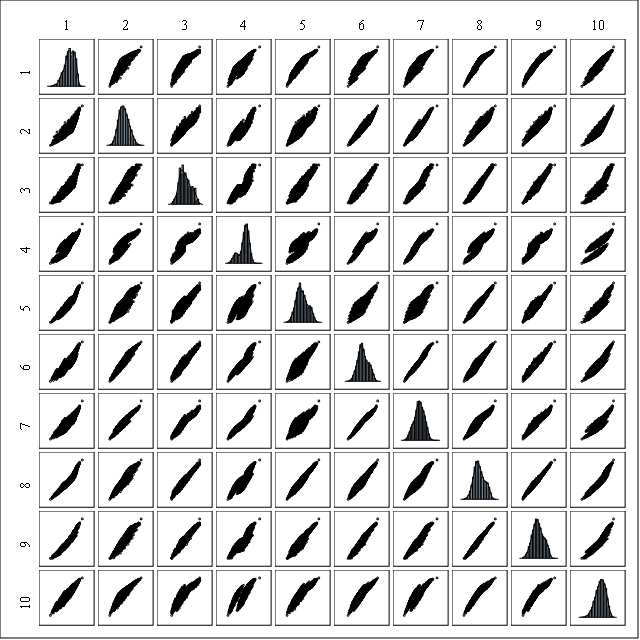

Supplement: Supplementary file 2 — Additional file 2. Scatter plot by country—Crosswalk sets. Denmark = ”1” France = ”2” Germany = ”3” Japan = ”4” Netherlands = ”5” Spain = ”6” Thailand = ”7” UK = ”8” US = ”9” Zimbabwe = ”10”. [file 13104_2019_4067_MOESM2_ESM.tif]
